# Test-Retest Reliability of Audiometric Assessment in Individuals With Mild Dementia

**DOI:** 10.1001/jamaoto.2021.0012

**Published:** 2021-03-04

**Authors:** Katrina S. McClannahan, Yi-Fang Chiu, Mitchell S. Sommers, Jonathan E. Peelle

**Affiliations:** 1 Department of Psychological and Brain Sciences, Washington University in St Louis, St Louis, Missouri; 2 Department of Communication Sciences and Disorders, St Louis University, St Louis, Missouri; 3 Department of Otolaryngology, Washington University in St Louis, St Louis, Missouri

## Abstract

**Importance:**

Accurate assessment of hearing is critically important regardless of a person’s cognitive ability. The degree to which hearing can be reliably measured in adults with mild dementia has not been determined.

**Objective:**

To obtain quantitative measures of reliability to evaluate the degree to which audiologic testing can be accurately conducted in older adults with mild dementia.

**Design, Setting, and Participants:**

This repeated-measures cross-sectional study consisted of a comprehensive audiologic assessment on 2 occasions separated by 1 to 2 weeks performed in the department of otolaryngology at the Washington University School of Medicine from December 3, 2018, to March 4, 2020. Participants were 15 older adults with a verified diagnosis of mild dementia and 32 older adults without a verified diagnosis of mild dementia who were recruited from the Knight Alzheimer Disease Research Center at Washington University in St Louis.

**Main Outcomes and Measures:**

Test-retest reliability was assessed for tympanometry, acoustic reflex thresholds, otoacoustic emissions, hearing sensitivity, speech reception threshold, speech perception in noise, and hearing handicap, using standard clinical audiology measures.

**Results:**

A total of 47 older adults (26 women; mean [SD] age, 74.8 [6.0] years [range, 53-87 years]), including 32 with normal cognitive function and 15 with very mild or mild dementia, completed the study protocol. For participants with mild dementia, high test-retest reliability (Spearman ρ > 0.80) was found for most measures typically included in a comprehensive audiometric evaluation. For acoustic reflex thresholds, agreement was moderate to high, averaging approximately 83% across frequencies for both groups. Scores for the screening Hearing Handicap Inventory for the Elderly at time 1 and time 2 were highly correlated for the group with normal cognitive function (*r* = 0.84 [95% CI, 0.70-0.93]) and for the group with mild dementia (*r* = 0.96 [95% CI, 0.88-0.99]). For hearing thresholds, all rank-order correlations were above 0.80 with 95% CIs at or below 15% in width, with the exception of a moderate correlation of bone conduction thresholds at 500 Hz for the group with normal cognitive function (*r* = 0.69 [95% CI, 0.50-0.84]) and slightly wider 95% CIs for low-frequency bone conduction thresholds for both groups. For speech reception thresholds, correlations were high for groups with normal cognitive function (*r* = 0.91 [95% CI, 0.84-0.95]) and mild dementia (*r* = 0.83 [95% CI, 0.63-0.94]).

**Conclusions and Relevance:**

Test-retest reliability for hearing measures obtained from participants with mild dementia was comparable to that obtained from cognitively normal participants. These findings suggest that mild cognitive impairment does not preclude accurate audiologic assessment.

## Key Points

**Question:**

Are the measures used in standard hearing assessments reliable in individuals with mild dementia?

**Findings:**

This cross-sectional study that included 15 adults with mild dementia and 32 adults with normal cognitive function found high test-retest reliability for hearing thresholds, hearing handicap assessment, and several physiological measures typically included in a standard audiology test battery.

**Meaning:**

This study suggests that hearing assessments completed under ideal conditions are reliable in adults with mild dementia.

## Teaser

This cross-sectional study obtains quantitative measures of reliability to evaluate the degree to which audiologic testing can be accurately conducted for older adults with mild dementia.

## Introduction

Age-related hearing loss is highly prevalent and has far-reaching health, emotional, and social consequences.^1,2^ Importantly, age-related hearing loss is underdiagnosed and undertreated.^3,4^ With dementia most frequently affecting adults older than 60 years, there is a sizable population of older adults who are experiencing both dementia and age-related hearing loss. Listening to and understanding speech, particularly in noisy environments, can be frustrating, effortful, and often impossible for individuals with age-related hearing loss. For listeners who are also experiencing pathologic changes in cognition associated with dementia, these situations may be even more discouraging and difficult to manage. Because of the potential for increased communicative difficulty for adults with concurrent age-related hearing loss and dementia, and a possible association between hearing loss and cognitive decline,^5,6^ there is a critical need to establish the accuracy of hearing measurements in listeners with dementia.

Assessment of hearing loss typically includes a battery of physiological and behavioral measures with results of outer, middle, and inner ear health that audiologists use to inform individualized treatment plans. Several measures do not require active participation by the patient, such as middle ear testing via tympanometry and acoustic reflexes and inner ear assessment via otoacoustic emissions. However, because hearing is a perceptual process, the criterion standard for the assessment of hearing sensitivity—audiometry—requires active and interactive behavioral responses from patients.

In listeners without dementia, the pure-tone audiogram is generally presumed to have good reliability.^7,8,9^ However, it has long been appreciated that individual differences in cognitive abilities may affect test results.^10^ For example, in pediatric audiology, clinicians significantly modify standard test procedures in anticipation of children’s short attention spans and developmental limitations. Visual reinforcement audiometry (for infants and toddlers) and conditioned play audiometry (for children between 2 and 5 years of age) use toys and conditioned responses in place of standard “hand raise” voluntary responses. In addition, making the test more dynamic by switching ears often and prioritizing testing from 500 to 4000 Hz increases children’s engagement and provides clinicians with information about hearing sensitivity most important for speech.^11^ Furthermore, the association between acoustic challenge and cognitive effort^12^ raises the concern that listeners with both hearing loss and cognitive difficulty may struggle to adequately perform auditory tasks involving decision-making.

Studies evaluating the association between hearing loss and dementia typically quantify hearing abilities using self-reported hearing measures^13,14^ or standard pure-tone audiometry.^6,15^ However, to our knowledge, there is a lack of information regarding how dementia may affect the ability to accurately assess hearing.^16^ Self-reported measures, which are only moderately correlated with hearing thresholds,^17^ may not be ideal for listeners with dementia, as previous studies indicate that reliable self-reported health measures may be difficult to obtain in this population.^18^ It is also possible that standard pure-tone audiometry might be challenging for individuals with dementia because difficulty following instructions, increased irritability, and memory deficits are hallmarks of cognitive decline.

In this context, then, it is important to assess whether individuals with dementia, who may be experiencing a decline in memory and introspection as well as increased agitation and confusion, might also struggle to reliably respond during hearing assessments that require attentive behavioral responses. To address these issues, we examined the test-retest reliability of several audiologic measures in listeners with normal cognitive function and with mild dementia. We hypothesized that mild dementia would negatively affect the reliability of pure-tone audiometry, speech in quiet thresholds, speech in noise thresholds, and self-reported hearing handicap, all of which require active participation. Our goal was to obtain quantitative measures of reliability to evaluate the degree to which audiologic testing can be accurately conducted in older adults with mild dementia.

## Methods

### Participants

Participants were recruited from the Charles F. and Joanne Knight Alzheimer Disease Research Center at the Washington University School of Medicine, St Louis, Missouri. Participants completed written informed consent and were paid $90 for their time. The study was approved by the Washington University Institutional Review Board.

**Figure 1.**
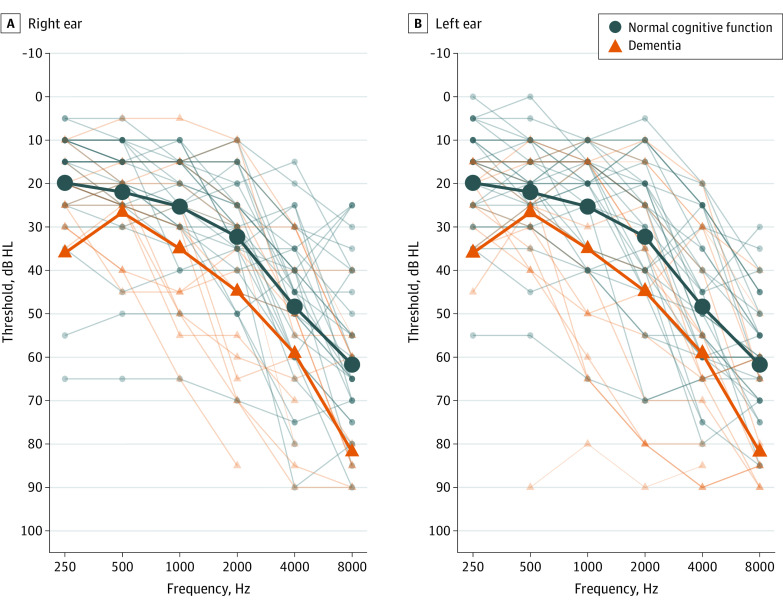
Individual and Group Mean Left and Right Ear Pure-Tone Air Conduction Thresholds at First Visit Thin lines indicate individual participant thresholds, and thick lines indicate group mean values. HL indicates hearing level.

Prior to this study, participants were evaluated at the Knight Alzheimer Disease Research Center and assigned Clinical Dementia Ratings (CDRs) through structured interviews with the participant and their coparticipant (generally a spouse or caregiver). The CDR Dementia Staging Instrument classifies dementia severity on a 5-point scale (normal cognition, 0; very mild dementia, 0.5; mild dementia, 1; moderate dementia, 2; and severe dementia, 3).^19,20^ The mean (SD) amount of time between CDR assessment and participation in the study was 9.1 (4.3) months (range, 1-27 months). Participants with neurologic diagnoses other than Alzheimer disease dementia or who were unable to read a computer screen with corrected vision were excluded. Figure 1 shows air conduction thresholds.

### Procedure

The following measures were completed from December 3, 2018, to March 4, 2020, at 2 sessions (time 1 and time 2) separated by 7 to 14 days (mean [SD], 8.2 [2.6] days). Testing time was limited to 1 hour and 20 minutes. With the exception of the screening hearing handicap survey, which was completed at the beginning of each session, all physiological measures were completed prior to behavioral measures and in the order listed below. All measures were administered in a sound-attenuating booth.

#### Physiological Measures

##### Otoscopy

Otoscopy was used to assess the presence of obstructing cerumen or abnormal tympanic membrane appearance. A cerumen impaction was identified in 1 participant who was referred to their primary care physician for cerumen management. They were rescheduled, and on return, otoscopy revealed clear canals bilaterally.

##### Tympanometry

Left and right ear tympanogram peak pressure, peak compensated static acoustic admittance, and equivalent ear canal volume were measured with an Interacoustics Titan impedance module (Interacoustics A/S), using a standard 226-Hz probe tone.

##### Distortion Product Otoacoustic Emissions

Left and right ear distortion product otoacoustic emissions were obtained using the Interacoustics Titan distortion product otoacoustic emission module. Emissions were measured at 0.75, 1, 2, 3, 4, 6, and 8 kHz with a tone level of 65 dB sound pressure level and an F2 to F1 ratio of 1.22. Responses were considered present if the response level was −10 dB or more and the signal to noise ratio was more than 7.

##### Acoustic Reflex Thresholds

Ipsilateral and contralateral left and right ear acoustic reflex thresholds were obtained at 0.5, 1, 2, and 4 kHz with the Interacoustics Titan impedance module. A reflex was considered present if the downward deflection reached 0.02 mL at a given frequency.

#### Behavioral Measures

##### Hearing Handicap

Participants completed the screening version of the Hearing Handicap Inventory for the Elderly,^21,22^ consisting of 10 questions regarding the impact of perceived hearing loss on daily communicative function. Participants answered never (score, 0), sometimes (score, 2), or always (score, 4) to questions such as “Does a hearing problem cause you to feel embarrassed when meeting new people?” The possible total score range was 0 (no hearing handicap) to 40 (significant hearing handicap).

##### Audiometric Assessment

Pure-tone thresholds were obtained using the modified Hughson-Westlake procedure^23^ with a GSI 61 audiometer or Audiostar Pro audiometer (Grason-Stadler). We modified the standard audiometric test order based on pediatric audiology best practices.^11^ Specifically, testing began in the participant’s better ear (if one was specified) at 2 kHz. After a threshold was established for the better ear, the poorer ear was tested at the same frequency. This procedure was followed for subsequent frequencies, in the following order: 0.5, 1, and 4 kHz (see Table 1 for complete protocol and test order).

**Table 1.** Audiologic Measures and Order of Test Battery

Measure	Deviations from standard adult audiometry
Air conduction thresholds	“Better ear” first, followed by the other ear at each frequency, before moving on; tone order: 2, 0.5, 1, and 4 kHz; masking when required
Speech reception thresholds	None
Bone conduction thresholds	None
Quick Speech perception in noise test	Diotic presentation
Air conduction thresholds^a^	Fill in audiogram, time permitting; tone order: 3, 6, 8, 0.25 kHz and interoctaves if ≥20 dB HL difference at adjacent frequencies; masking when required

Abbreviation: HL, hearing level.

^a^ Tests appear in the order they were conducted; an abbreviated number of air conduction thresholds were measured at the beginning of the session, followed by speech reception thresholds, bone conduction thresholds, and a speech perception in noise measure. The remainder of the audiogram was then filled in, time permitting.

##### Speech Reception Threshold

Speech reception thresholds were obtained for left and right ears using standard clinical procedures.^24^ In brief, individuals were familiarized with CID (Central Institute for the Deaf) W-1 spondee words using recorded materials (8 participants, all CDR 0) or with monitored live voice (39 participants).^25^ Speech reception thresholds were then obtained first for the better ear and then for the poorer ear.

##### Speech Perception in Noise

Speech perception in noise was assessed using the Quick Speech in Noise Test (QuickSIN; Etymotic Research). Materials were routed to the audiometer from an external compact disc player (GSI 61) or integrated with the audiometer (Audiostar Pro). All materials were calibrated based on manufacturers’ specifications. Two QuickSIN lists were presented at each session (time 1, lists 1 and 2; and time 2, lists 3 and 4). Presentation levels were based on the instructions from the test manual. In brief, for participants with a better ear pure-tone average of 45 dB in hearing level (HL) or lower, 70 dB HL was used as the presentation level. This level was adjusted upward to a perceptual “loud but comfortable” level above 70 dB HL for participants with a pure-tone average of 50 dB HL or higher. Signal to noise ratio loss was estimated for each participant by averaging the scores for the 2 lists.

### Statistical Analysis

Data and analysis scripts are available online.^26^ Data were analyzed in R, version 3.6.3 (R Foundation for Statistical Computing).^27^ Data were checked for normality and outliers, defined as scores more than 1.5 times the interquartile range. Analyses were conducted with and without outliers, and outliers are described for each measure in the sections below. With the exception of the QuickSIN speech perception in noise measure, there were no significant changes in overall results with outliers removed, and they were subsequently retained in the final analyses. QuickSIN results are reported with outliers removed. Changes in variances and mean values from time 1 to time 2 for each group were assessed for each measure. Test results were not significant at *P* < .05 with the exception of tympanogram peak pressure, which showed significant differences in variance from time 1 to time 2 for both groups.^26^

Because of deviations from normality, Spearman rank-order correlations were used for all analyses. To further evaluate the precision of the reliability estimates, 95% CIs were calculated for each measure using bootstrap resampling (implemented in the rcompanion package^28^). As is commonly reported, we treated left ear and right ear measures as being independent. However, because left and right ear hearing sensitivity measures are in fact correlated, we conducted follow-up analyses on single-ear data; in all cases, the single-ear results were compatible with the combined data.^26^ For measures analyzing left and right ears independently, the sample included 64 ears in the group with normal cognitive function and 30 ears in the group with mild dementia, unless otherwise specified.

## Results

A total of 47 older adults (mean [SD] age, 74.8 [6.0] years [range, 53-87 years]), including 32 (18 women) with normal cognitive function (CDR 0), and 15 (8 women) with a diagnosis of very mild or mild dementia (CDR 0.5 and 1; further referred to as “mild dementia”) completed the study protocol. All participants completed both sessions within the allotted time, with the exception of 1 individual in the group with mild dementia. This participant required frequent task reinstruction. As a result, the final 2 (8 and 0.25 kHz) air conduction thresholds were not assessed owing to time constraints at time 1. However, the participant completed all measures within the allotted time at time 2.

### Physiological Measures

#### Tympanometry

Measures at time 2 were not obtained for 2 ears in the group with mild dementia, 1 owing to inability to maintain a seal and 1 with no identifiable peak in pressure or admittance (without cerumen obstruction). Subsequently, 28 ears were included in the correlations for peak pressure, 28 for static admittance, and 29 for ear canal volume.

Ear canal volume and static admittance at time 1 and time 2 were highly correlated for both groups, with Spearman ρ > 0.80 for each correlation and with 95% CI widths between 10% and 20% around the point estimates. However, peak pressure from time 1 to time 2 was weakly correlated for the group with normal cognitive function (*r* = 0.35 [95% CI, 0.06-0.59]) and moderately correlated for the group with mild dementia (*r* = 0.64 [95% CI, 0.28-0.86]). Although correlations were not high and there were significant differences in variance from time 1 to time 2 for each group (as previously discussed), all ears were within clinically defined normal limits (−150 to 25 daPa) with the exception of 6 ears in the control group that were only slightly outside that range.^26^

#### Acoustic Reflex Thresholds

One ear in the group with normal cognitive function was excluded from analysis of left ipsilateral and right contralateral reflexes at all frequencies owing to equipment malfunction at time 2. Responses were coded as present (1) and absent (0) to assess percentage agreement between time 1 and time 2. Percentage agreement was calculated for left and right ear, ipsilateral and contralateral thresholds at each frequency. Agreement was moderate to high, averaging approximately 83% across frequencies for both groups (Table 2).

**Table 2.** Mean Percentage Agreement for Acoustic Reflex Thresholds and Distortion Product Otoacoustic Emissions

Stimulus ear	Probe ear	Acoustic reflex thresholds, mean agreement, %				Ear	Distortion product otoacoustic emissions, mean agreement, %						
		500 Hz	1000 Hz	2000 Hz	4000 Hz		750 Hz	1000 Hz	2000 Hz	3000 Hz	4000 Hz	6000 Hz	8000 Hz
Control													
Left	Left	90.3	80.6	87.1	80.6	Left	78.1	81.3	81.3	84.4	96.9	100	100
Left	Right	84.4	71.9	81.3	81.3								
Dementia													
Left	Left	80.0	86.7	93.3	80.0	Left	80.0	93.3	93.3	100	100	100	100
Left	Right	80.0	86.7	93.3	86.7								
Control													
Right	Right	78.1	81.3	71.9	81.3	Right	81.3	81.3	75.0	81.3	96.9	93.8	100
Right	Left	87.1	93.5	87.1	87.1								
Dementia													
Right	Right	80.0	73.3	73.3	100.0	Right	86.7	100	93.3	100	93.3	100	100
Right	Left	66.7	80.0	80.0	80.0								

#### Distortion Product Otoacoustic Emissions

Responses were coded as present (1) and absent (0) to assess percentage agreement between time 1 and time 2. Percentage agreement was high for both groups, ranging from approximately 78% to 100%. The lowest agreement was seen in the low frequencies and likely reflects interference of low-frequency background noise (Table 2).

### Behavioral Measures

#### Hearing Handicap

Scores for the screening Hearing Handicap Inventory for the Elderly at time 1 and time 2 were highly correlated for the group with normal cognitive function (*r* = 0.84 [95% CI, 0.70-0.93]) and for the group with mild dementia (*r* = 0.96 [95% CI, 0.88-0.99]), indicating good test-retest reliability.

#### Hearing Thresholds

Thresholds for the left and right ears were treated as independent. Mean air conduction thresholds for low- (0.25, 0.5, and 1 kHz), middle- (2, 3, 4 kHz), and high-frequency (6 and 8 kHz) ranges, as well as bone conduction thresholds (0.5, 1, 2, and 4 kHz), were used in the analysis. One participant with normal cognitive function had asymmetric hearing loss with no measurable hearing in 1 ear; that ear was excluded from all analyses. One ear in the group with normal cognitive function and 1 ear in the group with mild dementia were excluded from the air conduction high-frequency pure-tone average analysis owing to those 2 participants having no measurable hearing at 6 and 8 kHz. One ear in the group with normal cognitive function was excluded from bone conduction for 1 and 4 kHz owing to failure to mask and no response at system limits, respectively. One, 2, and 5 ears in the group with mild dementia were excluded from bone conduction for 0.5, 2, and 4 kHz, respectively, owing to no response at system limits (7 ears) and failure to mask (1 ear).^26^

**Figure 2.**
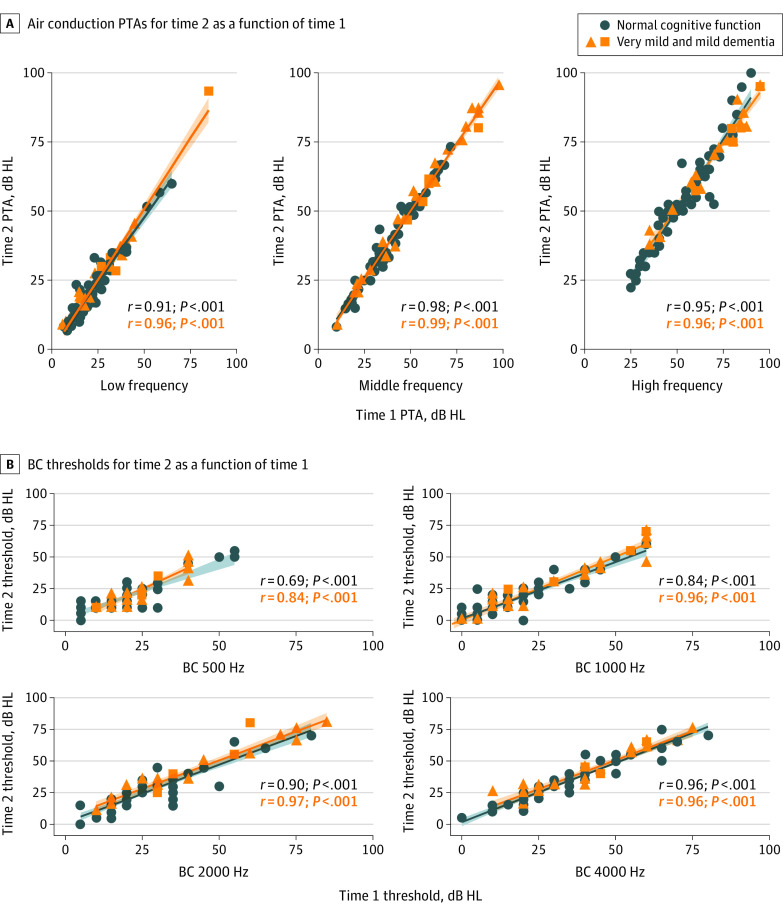
Hearing Sensitivity Scatterplots A, Low- (250, 500, and 1000 Hz), middle- (2000, 3000, and 4000 Hz), and high-frequency (6000 and 8000 Hz) pure-tone air conduction threshold averages for time 2 as a function of time 1. B, Bone conduction (BC) thresholds at 500, 1000, 2000, and 4000 Hz for time 2 as a function of time 1. HL indicates hearing level; PTA, pure-tone average. Shaded areas around lines indicate 95% CIs.

Scatterplots showing low-, middle-, and high-frequency air conduction thresholds and bone conduction thresholds at time 2 as a function of time 1 are shown in Figure 2. All rank-order correlations were above 0.80 with 95% CIs at or below 15% in width, with the exception of a moderate correlation of bone conduction thresholds at 500 Hz for the group with normal cognitive function (*r* = 0.69 [95% CI, 0.50-0.84]) and slightly wider 95% CIs for low-frequency bone conduction thresholds for both groups. These results indicate good test-retest reliability of hearing thresholds for both groups. The mean dB HL difference between time 1 and time 2 was under 5 dB at all frequencies for air and bone conduction thresholds, for both groups.^26^

#### Speech Reception Thresholds

**Figure 3.**
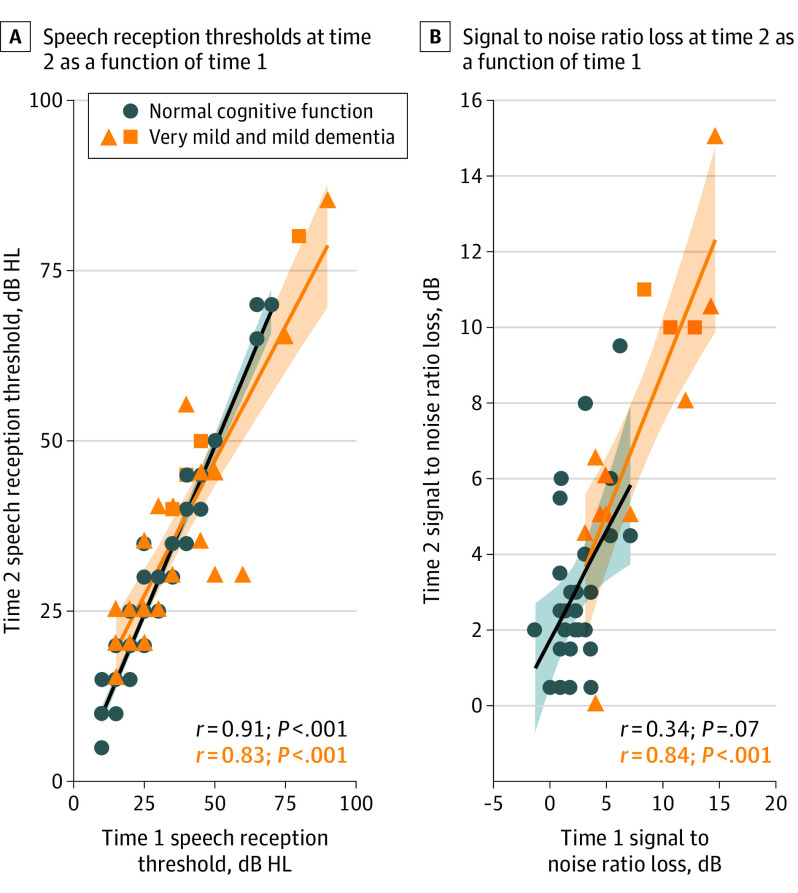
Speech Measures Scatterplots A, Speech reception thresholds at time 2 as a function of time 1. B, Signal to noise ratio loss at time 2 as a function of time 1. HL indicates hearing level. Shaded areas around lines indicate 95% CIs.

Speech reception thresholds for left and right ears were treated as independent. Raw data, in dB HL, were used for analysis. Correlations were high for groups with normal cognitive function (*r* = 0.91 [95% CI, 0.84-0.95]) and mild dementia (*r* = 0.83 [95% CI, 0.63-0.94]), indicating good test-retest reliability for both groups (Figure 3A).

#### Speech Perception in Noise

Four participants in the group with normal cognitive function were excluded from analysis owing to equipment malfunction at time 2 (1 participant) and having QuickSIN scores identified as outliers at time 1 or time 2 (3 participants).^26^ With outliers excluded, the correlation between time 1 and time 2 QuickSIN scores was not significant for the group with normal cognitive function (*r* = 0.34 [95% CI, −0.08 to 0.68]) but was high for the group with mild dementia (*r* = 0.84 [95% CI, 0.53-0.96]). The results observed for the group with normal cognitive function are likely due to restricted variance (see clustering of scores between 0 and 2.5 dB in Figure 3B).

## Discussion

The goal of this study was to assess the reliability of standard audiologic measures for individuals with mild dementia. We anticipated that mild dementia might negatively affect the reliability of pure tone, speech in quiet and speech in noise thresholds, and self-reported hearing handicap, which all require active participation for testing and could be affected by the cognitive consequences of mild dementia. We found test-retest reliability to be comparably high between the groups with and without mild dementia. Although our sample size was limited and the results should be interpreted with caution, the 95% CIs surrounding the reliability point estimates were generally in the range of 10% to 20%, meaning that we expect larger samples to show similar reliability. Our results suggest that interactions between hearing loss and mild dementia do not appear to affect the accurate assessment of key measures of hearing sensitivity, auditory function, and self-reported hearing handicap.

We maximized best-practice procedures for hearing assessment in this protocol in several ways. All measures were performed in a sound-attenuating booth with good lighting and little visual or auditory distractors. The accuracy and reliability of assessments in noisier or more visually intrusive environments, however, may be significantly diminished. In addition, we reorganized the standard audiologic test order, prioritizing ear-specific thresholds at an abbreviated number of frequencies. It is possible that reordering frequencies increased the salience of the pure tones by frequently switching from ear to ear and roving in frequency. Reliability may be reduced when standard protocols assess a single ear at a time for all frequencies and ascend or descend in frequency in predictable octave steps. The potential consequences of these changes require further examination.

Although some prior studies suggested difficulties assessing hearing sensitivity in individuals with dementia,^16^ many included participants with moderate to severe dementia. We limited our focus to those with mild dementia (CDR 0.5 and 1) and have identified hearing assessments to be reliable for individuals in the early stages of dementia progression. Our findings emphasize the importance of hearing loss screening and diagnosis for older adults before dementia symptoms become more severe, at which time an individual’s ability to participate in hearing assessments may be affected. Future studies are needed to address how more severe dementia symptoms may affect the accuracy and reliability of audiologic assessments.

### Limitations

The sample size was limited, so our results should be interpreted with caution; however, we are confident that results would be comparable in a larger sample. The current study only measured the reliability of hearing measures for adults with mild dementia. Therefore, this study does not provide a complete picture of how symptoms of more advanced dementia may interact with the ability to assess hearing. Future studies are warranted.

## Conclusions

In this study, mild dementia and associated symptoms did not appear to affect the reliability of measures commonly included in a standard audiometric test battery, when performed under ideal conditions. Observing no significant group differences may initially seem less compelling than finding large group differences. However, we argue that our findings are as powerful, if not more so, than if we would have found reduced reliability for the dementia group. There have been many studies that have used audiometry and self-reported hearing measures to examine the association between hearing loss and dementia. The accuracy of these findings is predicated on the assumption that the measures are reliable for those participants. Given that audiometry requires participants to engage their attention, executive function, and short-term memory, all of which are specifically affected by cognitive changes associated with mild or worse dementia, it is essential to establish the measures’ reliability. Our findings support the continued use of these measures for adults with mild dementia, for both clinical and research purposes.

Future studies need to address the possibility that, as dementia symptoms worsen, it may become more difficult to administer auditory tests, particularly those that require active participation. We hope that our findings also encourage other researchers to consider reliability in a variety of populations and set an example for how testing and measurement might be approached. Having confidence in the measures used to assess auditory function in adults with dementia is critical for clinicians, in order to provide optimal hearing health care, and for researchers, when examining the potential link between dementia and hearing loss to ensure the association between the two is not confounded by poor test reliability.
